# Investigating the Impact of Co-processed Excipients on the Formulation of Bromhexine Hydrochloride Orally Disintegrating Tablets (ODTs)

**DOI:** 10.1007/s11095-023-03605-x

**Published:** 2023-09-19

**Authors:** Krzysztof Woyna-Orlewicz, Witold Brniak, Wiktor Tatara, Magdalena Strzebońska, Dorota Haznar-Garbacz, Joanna Szafraniec-Szczęsny, Agata Antosik-Rogóż, Kamil Wojteczko, Mirosław Strózik, Mateusz Kurek, Renata Jachowicz, Aleksander Mendyk

**Affiliations:** 1 Department of Pharmaceutical Technology and Biopharmaceutics, Faculty of Pharmacy, Jagiellonian University Medical College, Ul. Medyczna 9, 30-688, Krakow, Poland; 2 F1Pharma S.A, Ul. Bobrzynskiego 14, 30-348 Krakow, Poland; 3 Department of Environmental Protection, Faculty of Geology, Geophysics and Environmental Protection, AGH University of Science and Technology, Al. Mickiewicza 30, 30-059 Kraków, Poland; 4 Department of Drug Form Technology, Wroclaw Medical University, Wrocław, Poland; 5 CHDE Polska S.A, Biesiadna 7, 35-304, Rzeszow, Poland; 6 Department of Ceramics and Refractories, AGH University of Science and Technology, 30-059, Krakow, Poland

**Keywords:** compactability, compressibility, co-processed excipients, direct compression, disintegration time, mechanical properties, orally disintegrating tablets, orodispersible tablets, superdisintegrant, tablet ability

## Abstract

**Purpose:**

Orodispersible tablets (orally disintegrating tablets, ODTs) have been used in pharmacotherapy for over 20 years since they overcome the problems with swallowing solid dosage forms. The successful formula manufactured by direct compression shall ensure acceptable mechanical strength and short disintegration time. Our research aimed to develop ODTs containing bromhexine hydrochloride suitable for registration in accordance with EMA requirements.

**Methods:**

We examined the performance of five multifunctional co-processed excipients, i.e., F-Melt® C, F-Melt® M, Ludiflash®, Pharmaburst® 500 and Prosolv® ODT G2 as well as self-prepared physical blend of directly compressible excipients. We tested powder flow, true density, compaction characteristics and tableting speed sensitivity.

**Results:**

The manufacturability studies confirmed that all the co-processed excipients are very effective as the ODT formula constituents. We noticed superior properties of both F-Melt’s®, expressed by good mechanical strength of tablets and short disintegration time. Ludiflash® showed excellent performance due to low works of plastic deformation, elastic recovery and ejection. However, the tablets released less than 30% of the drug. Also, the self-prepared blend of excipients was found sufficient for ODT application and successfully transferred to production scale. Outcome of the scale-up trial revealed that the tablets complied with compendial requirements for orodispersible tablets.

**Conclusions:**

We proved that the active ingredient cannot be absorbed in oral cavity and its dissolution profiles in media representing upper part of gastrointestinal tract are similar to marketed immediate release drug product. In our opinion, the developed formula is suitable for registration within the well-established use procedure without necessity of bioequivalence testing.

**Supplementary Information:**

The online version contains supplementary material available at 10.1007/s11095-023-03605-x.

## Introduction

Orodispersible tablets or orally disintegrating tablets (ODT) are uncoated, single-unit dosage forms intended to be placed in the mouth, where they disperse in saliva before being swallowed [1]. They have been used in pharmacotherapy for over 20 years, gaining more and more importance and being significantly upgraded [2–5]. The main reason for their application is to overcome problems with swallowing solid dosage forms (medication dysphagia), which affects from 10 to 60% of all patients, depending on the investigated population, and is noticeably higher among children and elderly [6, 7].

The earliest technology used in ODTs formulation was freeze drying, patented in 1973 under the tradename LYOC® [8]. However, the first registered drug product (Pepcidin Rapitab) was introduced to the market 20 years later and produced with Zydis® technology [9]. Since then, many other methods were used to manufacture ODTs, including casting/molding or direct compression [2, 3, 5]. Nowadays, ODTs are available not only as simple immediate release formulations, but also as advanced dosage forms with incorporated microcapsules, microspheres, pellets, or granules characterized by taste masking, prolonged or delayed release properties. These advanced systems are sometimes called “3^rd^ generation” or “new generation ODTs” [10]. What is more, in 2015, the first 3D-printed ODTs were produced with the ZipDose technology and marketed by Aprecia Pharmaceuticals under the tradename Spritam® (levetiracetam) [11]. The main advantage of this ODTs, containing up to 1000 mg of API, is an extremely short disintegration time (2—27 s) reported in *in vivo* tests, which complies with both USP and European Pharmacopoeia. Furthermore, in 2016, the first ODT formulation with prolonged release characteristics, Adzenys XR-ODT, was approved by Food and Drug Administration. It contains from 3.1 to 18.8 mg amphetamine encapsulated in two different kinds of microparticles compressed into ODT: the half dose of the drug substance is released immediately after dispersion, while the rest of it for the next 24 h [12, 13].

Most of the currently marketed ODTs are produced with direct compression method [5], which is widely used in the manufacturing the conventional tablets due to the overall simplicity, ease of the upscaling of the production process, and excellent cost- effectiveness. Compressed tablets usually have better mechanical properties and more resistance to humidity than ODTs produced with other methods. Thus, their packaging and storage is much easier and less expensive (it may be even possible to store some products in multi-unit glass bottles or plastic containers). On the other hand, their disadvantage may be a longer disintegration time. However, the extensive development of novel superdisintegrants makes this less problematic. What is more, the production of ODTs becomes much easier due to the increasing availability of the co-processed excipients [14]. They are combinations of two or more excipients designed to physically modify their properties in a manner not achievable by simple physical mixing and without chemical change [15, 16]. They are produced with methods such as spray-drying, wet granulation, roller compaction, fluid bed spray granulation, melt granulation, roller drying, co-precipitation, co-transformation or milling [16]. Co-processing of excipients may improve their properties critical for direct compression, i.e., flowability by increasing the roundness of particles, compressibility by combination of materials with different plasticity and brittleness, and disintegration time by increasing the porosity of the material. The proper selection of one of them becomes crucial in the formulation process. However, so far, there are only a few papers published comparing properties of commercially available co-processed excipients. Therefore, evaluation of their effect on the ODTs characteristics may be challenging and time-consuming process [17–23].

The ODTs are designed to quickly disperse in the oral cavity, so it is possible that the active ingredient dissolves in saliva and is absorbed via the buccal mucosa [24]. Therefore, registration of ODT form as an alternative for existing immediate release (IR) product should be supported by bioequivalence studies. However, according to the EMA guidance [24] the ODT and IR products can be considered for a BCS based biowaiver if the applicant demonstrates that the active ingredient is not absorbed in the oral cavity [25].

Bromhexine is a synthetic derivative of an alkaloid obtained from the *Adhatoda vasica* plant. The substance has a secretory and secretomotor effect in the bronchial tree, which facilitates expectoration and relieves coughing [26] The indications for the use of bromhexine are acute and chronic bronchitis, bronchial asthma, cystic fibrosis, and postoperative respiratory rehabilitation. Mikhaylov *et al.* suggest that prophylactic treatment with the compound was associated with a reduced rate of symptomatic COVID-19 [27]. Bromhexine is quickly absorbed after oral administration. The maximum blood concentration occurs after about 1 h. The half-life of bromhexine is about 0.4 h. Bromhexine undergoes intensive metabolism in the liver, during which metabolites are formed, including pharmacologically active ambroxol. The drug is characterized by low toxicity. Bromhexine has no carcinogenic effect, no mutagenic or teratogenic properties, and it does not affect reproductive capacity. Bromhexine is usually used in the amount of 8 mg three times a day [26, 28]. The products containing bromhexine hydrochloride are used in treatment for decades, and their efficacy and safety are thoroughly documented and available by means of scientific bibliography. Therefore, the drug fulfils well-established medicinal use criteria according to EU regulations [29, 30].

The aim of our study was to investigate the effect of multifunctional co-processed excipients, i.e., F-Melt^®^ C, F-Melt^®^ M, Ludiflash^®^, Pharmaburst^®^ 500, Prosolv^®^ ODT G2, and self-developed physical blend of directly compressible excipients on the attributes of orodispersible tablets containing 8 mg of bromhexine hydrochloride. The studies include the investigation of the physical properties of the materials (SEM imaging, determination of particle size distribution, true density, loss on drying, flow and compaction characteristics, assessment of punch-displacement time profiles with identification of its plastic and elastic components, as well as maximum ejection force and work) and tablets, both placebo and drug-loaded. We characterized tablet’s attributes such as friability, resistance to crushing, mass uniformity, disintegration time, and dissolution performance. Selected formula was checked for robustness by transferring to a production scale. We tested drug release profiles and presented our opinion regarding possibility of registration of the drug product in European Union under the Well Established Use procedure.

## Materials and Methods

### Materials

All the co-processed excipients were kindly gifted by their manufacturers: F-Melt^®^ type C and M (Fuji Chemical Industries Co., Ltd., Osaka, Japan), Ludiflash^®^ (BASF, Ludwigshafen, Germany), Pharmaburst^®^ 500 (SPI Pharma, Wilmington, DE, USA) and Prosolv^®^ ODT G2 (JRS Pharma, Rosenberg, Germany). Ingredients for physical blend were purchased from their producers: mannitol – Pearlitol^®^ (Roquette, Lestrem, France), microcrystalline cellulose Avicel^®^ (FMC, Philadelphia, PA, USA), Kollidon^®^ CL (BASF, Ludwigshafen, Germany), Aerosil^®^ 200 (Evonik, Essen, Germany). Sodium stearyl fumarate – Novalube^®^, used as a lubricant, was provided from Nitika Pharmaceutical Specialities PVT. Ltd. (Nagpur, India). Bromhexine hydrochloride (BRX) was purchased from VenPetrochem (Mumbai, India). All chemicals were of pharmacopoeial or analytical grade.

### Characterization of Excipients

The evaluation of excipients included SEM imaging, measurements of particle size distribution, true density, loss on drying, flow and compaction characteristics, assessment of manufacturability (please see below). Qualitative compositions of the compared co-processed excipients are presented in Table I.

**Table I:** Composition of Evaluated Excipients [31]

Excipient	Ingredients	Manufacturer
F-Melt^®^ C	D-mannitol, microcrystalline cellulose, xylitol, crospovidone, dibasic calcium phosphate anhydrous	Fuji Chemical Industries
F-Melt^®^ M	D-mannitol, microcrystalline cellulose, xylitol, crospovidone, magnesium aluminometasilicates	Fuji Chemical Industries
Ludiflash^®^	D-mannitol, crospovidone, polyvinyl acetate	BASF
Pharmaburst^®^ 500	D-mannitol, sorbitol, precipitated silicon dioxide, crospovidone	SPI Pharma
Prosolv^®^ ODT G2	D-mannitol, microcrystalline cellulose, fructose and colloidal silicon dioxide, crospovidone	JRS Pharma
MIX	D-mannitol, microcrystalline cellulose, crospovidone, colloidal silica	N/A

The qualitative and quantitative composition of the MIX blend was based on the formulation experience of the team working on this project. The amounts of ingredients were adjusted in previous studies including commercial projects in industrial R&D lab.

#### Scanning Electron Microscopy (SEM) Imaging

Particle morphology was assessed using a PhenomPro desktop scanning electron microscope (Thermo Fisher Scientific, Waltham, MA, USA), equipped with a CeB6 electron source and a backscattered electron detector. The samples were placed on the conductive adhesive tape previously glued to a specimen mount. The excessive amount of powder was removed using a stream of nitrogen. The samples were measured without sputtering using a holder for non-conductive samples at acceleration voltage equal to 10 kV, and magnification equal to 300x.

#### Particle Size Analysis

The measurements of particle size distribution were performed using a Mastersizer 3000 equipped with an AeroS unit with high-energy venturi dispenser (Malvern Instruments, Malvern, United Kingdom) using a dry dispersion method. The vibrational plate frequency was adjusted to maintain the obscuration in the 0.5%—6% range. Samples of the excipients were measured using Fraunhofer approximation and the values of Dv_10_, Dv_50_, Dv_90_ and span were calculated. Each sample was measured six times (*n* = 6).

#### Flowability

Bulk and tapped densities were measured according to the European Pharmacopoeia (Ph. Eur.) monograph 2.9.34. Angle of repose, flow through orifice, compressibility index (Carr index) and Hausner ratio were established based on the monograph 2.9.36.

#### Loss on Drying

The loss of mass after drying was tested in line with Ph. Eur. monograph 2.2.32. by using HE53 Moisture Analyzer (Mettler Toledo, Greifensee, Switzerland).

#### True Density Measurement with gas Pycnometry

The true density (  ) of materials was determined by the helium pycnometry method using an AccuPyc II 1340 apparatus (Micromeritics Instruments, Norcross, GA, USA). Prior to the measurement samples were degassed under vacuum at room temperature. Each sample was measured with 20 purges. The arithmetic mean and standard deviations were calculated.

### Powder Compaction Analysis

Studies were conducted with a Gamlen D-Series compaction simulator (Gamlen Tableting Limited, Beckenham, United Kingdom). Samples of the excipients in the amount of 60 mg were weighted with a MS 105DU analytical balance (Mettler-Toledo, Greifensee, Switzerland) and compressed with a round, flat punch of 5 mm diameter moving with a constant speed of 60 mm/min until 60 kp load was reached. A linear compression and decompression phases, i.e. saw-tooth punch displacement–time profile were recorded 6 times for the each tested material [32]. Based on that, the force–displacement-time profiles were established. The own-developed software was used to measure values of plastic deformation work, elastic deformation work, flow work, ejection work and maximum ejection load.

### Preparation of Placebo Tablets

The co-processed excipients were blended for 5 min with 2% of sodium stearyl fumarate using cube mixer (Erweka, Langen, Germany) operated with 10 rpm speed. Quantity of the lubricant was in accordance with Brniak *et al.* findings [33]. The physical blend (MIX) was prepared in two stages. Firstly, D-mannitol, microcrystalline cellulose, crospovidone, and colloidal silica were mixed for 15 min using the cube mixer with a speed of 10 rpm. Then, 2% of sodium stearyl fumarate was loaded into the cube mixer and blending was continued for another 5 min. The blends were compressed into 500 mg placebo tablets of 12 mm diameter using EK0 single punch tablet press (Korsch, Berlin, Germany). Three compression forces were applied, i.e., 7.5 kN, 10 kN and 12.5 kN. The results were evaluated for manufacturability, tabletability, compressibility and compactibility.

### Preparation of Bromhexine Hydrochloride Tablets

In order to test the machine speed sensitivity, formulations containing 8 mg of bromhexine hydrochloride were prepared (Table II). The quantity of lubricant depended on recommendations of each co-processed excipient manufacturer and ranged from 1% to 2.5%. The own prepared blend was characterized by high ejection force/work (see Results Section), therefore the lubricant quantity was the highest (4%). Tablets were compressed with rotary tablet press (Korsch PH103, Berlin, Germany) equipped with flat-faced bevel-edged punches of 7 mm diameter. Ingredients were mixed in the cube mixer at the same parameters as placebo blends. Tablets were manufactured at two turret speeds, i.e., 38 rpm or 76 rpm, which resulted in a dwell times of 70 ms or 35 ms, respectively.

**Table II:** Composition of BRX Tablets

Composition of tablets [%]						
Bromhexine HCl (BRX)	8	8	8	8	8	8
F-Melt^®^ C	87	-	-	-	-	-
F-Melt^®^ M	-	87	-	-	-	-
Ludiflash^®^	-	-	86	-	-	-
Pharmaburst^®^ 500	-	-	-	85.5	-	-
Prosolv^®^ ODT G2	-	-	-	-	87	-
MIX	-	-	-	-	-	84
Sodium stearyl fumarate	1	1	2	2.5	1	4
Lemon flavor	2	2	2	2	2	2
Sucralose	2	2	2	2	2	2
Tablet mass (mg)	100	100	100	100	100	100

### Measurements of Tablet Attributes

#### Uniformity of Mass

Twenty tablets from each series were separately weighted with laboratory scale Vibra AJH-420CE (Shinko Denshi, Tokyo, Japan) and the uniformity of the mass was calculated according to the European Pharmacopoeia monograph 2.9.5 [1].

#### Resistance to Crushing (Hardness, Breaking Force) and Thickness

Resistance to crushing was measured according to Ph. Eur. 2.9.8. [1] with VK200 apparatus (Vankel VK 200, Varian Inc., Cary, NC, USA). The same apparatus was used for measurement of tablets thickness. Both were measured for 10 individual units pooled from every single lot. Tensile strength was calculated according to the equation:

$${T}_{s}= \frac{2F}{\pi Dh}$$

where F is the resistance to crushing of tablets [N],

D – tablet diameter [mm],

h – tablet thickness [mm].

#### Friability

The friability of tablets was determined according to the monograph 2.9.7. [1] with apparatus F1 (ZDMPF, Krakow, Poland). Each sample of at least 6.5 g was subjected to 100 cycles, after which the weight loss expressed in percent was calculated.

#### Disintegration Time

Disintegration time of tablets was tested with two methods: Ph. Eur. 2.9.1 [1] performed using a ZT72 apparatus (Erweka, Langen, Germany) with approximately 800 mL of distilled water at 37°C, and the innovative one that is considered biorelevant, utilizing BJKSN-13 apparatus [33]. The tablet was immersed in 5 mL of water at ca. 37°C and put under slight pressure by a rotating shaft imitating tongue [33].

#### Dissolution Studies

Dissolution of bromhexine hydrochloride under simulated gastrointestinal conditions was tested using a Ph. Eur. paddle apparatus operated at 50 rpm. We used 900 mL of 0.1 mol/L hydrochloric acid solution as the dissolution medium maintained at the temperature 37°C. Tablets made in production scale were additionally tested in 900 mL of phosphate buffer of pH = 4.5. Samples were collected after 5, 10, 15, 20, 30 and 45 min. During the following 15 min the paddles were operated at 200 rpm in the course of so-called infinity test. The dissolved active substance was quantified with HPLC method (see "Assay" Section).The dissolution rate of bromhexine hydrochloride under simulated oral conditions was tested in a Hanson Research Vision G2 Elite 8 paddle tester (Hanson Research, Chatsworth, CA, USA) equipped with small volume vessels. Six tablets were placed into 50 mL of artificial saliva of pH 6.8 at 37°C for 10 min [34]. The medium was stirred at 50 rpm.

#### Assay

The determination of assay of the active substance in BRX tablets and determination of API dissolved from tablets, was performed by high performance liquid chromatography (HPLC) using Hitachi Primaide MERCK and Hitachi Elite LaChrom, MERCK liquid chromatographs with DAD detector using VDSpher Pur C8-E column (4 µm, 150 × 3.9 mm). Chromatographic system: wavelength 248 nm, column temperature 35°C, autosampler temperature 20°C, flow rate 1.0 mL/min, time analysis 10 min. Mobile phase: methanol, acetonitrile and phosphate buffer in 40:20:40 ratio (v/v/v) adjusted to pH = 4.5 ± 0.05 with phosphoric acid 8.5% w/v. Both methods were validated [35].

### Scale-up of the Tableting Process

Two production lots were manufactured, each in the size of 540 000 tablets. The excipients were blended in a bin (L.B.Bohle, Ennigerloh, Germany) and compressed into tablets using rotary machine (Kilian S250 Smart, Germany). Following attributes of tablets were tested periodically: appearance, mass uniformity, hardness, thickness, friability, and disintegration time. Samples pooled at the beginning, middle and the end of the tableting process were additionally tested for content uniformity, assay, loss on drying, dissolution rate according to the methods described in "Measurements of tablet attributes" Section. At the end of the process yield was checked.

### Statistical Analysis

Measurements were evaluated with normal distribution measures like average, range, standard deviation, coefficient of variation (RSD) and confidence intervals (95%). The groups of results were compared with one-way ANOVA and the post-hoc Sheffe test. The significance level was set at p < 0.05. The calculations were performed by using Tibco^®^Statistica™ (Tibco Software, Palo Alto, CA, USA).

## Results and Discussion

### Properties of the Materials

#### Characteristic of Powders

**Fig. 1:**
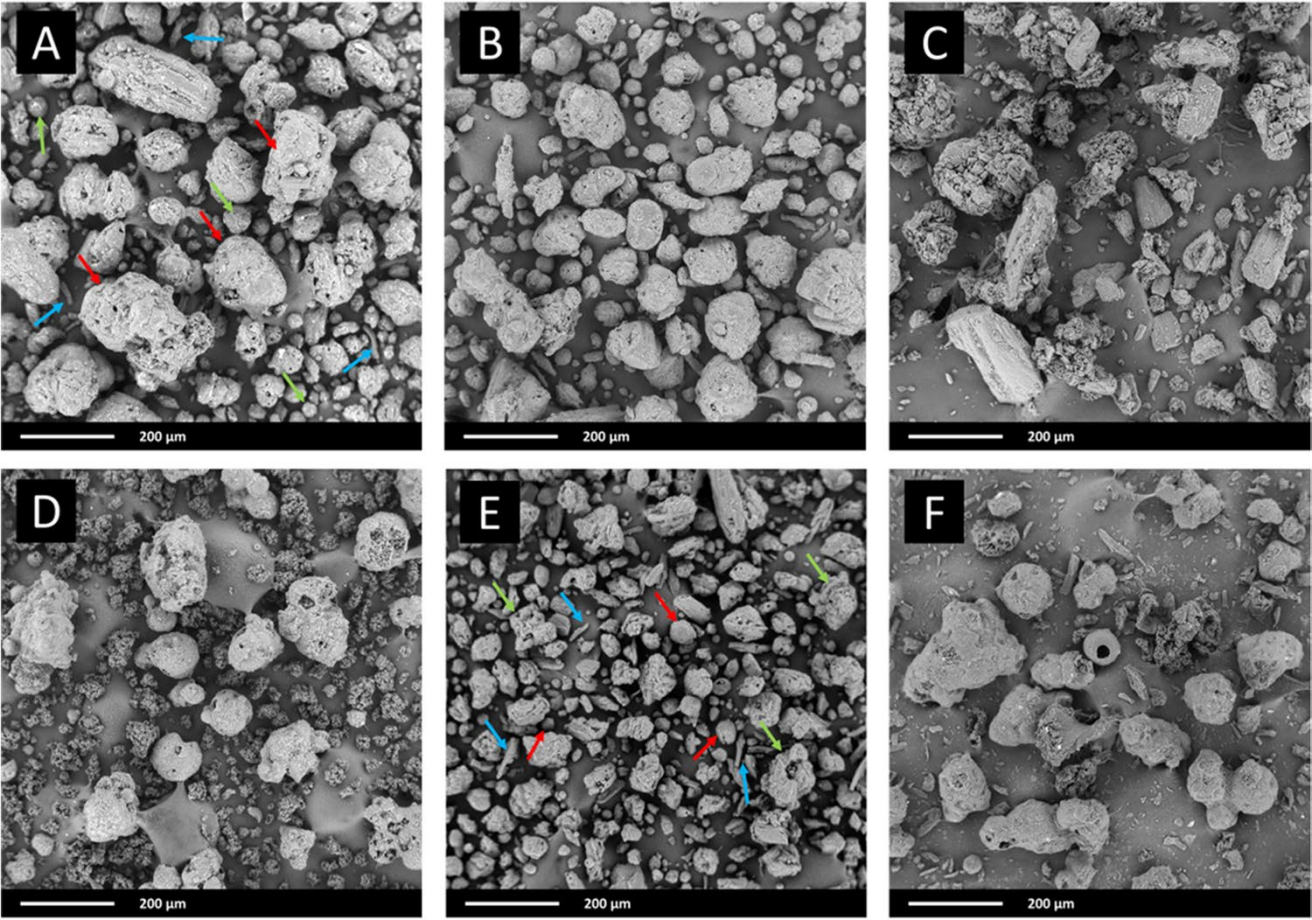
SEM images of F-Melt type C (**A**), F-Melt type M (**B**), Ludiflash (**C**), Pharmaburst 500 (**D**), Prosolv ODT G2 (**E**), and authors mix (**F**).

The analysis of SEM pictures revealed that the sample of F-Melt^®^ C contains three types of particles (Fig. 1A). The most noticeable are spherical (red arrows) of diameter between ca. 80—200 µm. Their surfaces are smooth with some irregularities. The second type is smooth granules of diameter not exceeding 100 µm (green arrows), while the third one consists of elongated, rod-like particles of length below 100 µm (blue arrows). The alike microstructure was noticed for F-Melt^®^ M (Fig. 1B), which probably results from a similar manufacturing method applied by Fuji Chemical Industries. Particle size distributions (Fig. S1) of both F-Melts^®^ are also similar, each having only one maximum and tailing towards small particles. However, the distribution of F-Melt^®^ M is a bit wider than of F-Melt^®^ C (distribution spans are equal to 1.77 and 1.38, respectively) (Fig. S1). According to Ph. Eur. 2.9.36, flow properties of the F-Melts are good in expression of Carr index, Hausner ratio and angle of repose (Tab.4).

Ludiflash^®^ particles are the biggest ones, reaching 300 µm (Fig. 1C). The distribution of their size is the widest among the tested samples, with one maximum, long left-sided tail, and the highest distribution span (above 2.5) (Table III, Fig. S1). The flowability measures indicate fair flow of this excipient (Table IV).

**Table III:** Excipients’ Particle Sizes and Distribution Span Obtained in Laser Diffraction Measurements

Excipient	Particle size [μm]			Span
	Dv_10_	Dv_50_	Dv_90_	
F-Melt^®^ C	61	139	253	1.38
F-Melt^®^ M	56	140	304	1.77
Ludiflash^®^	36	110	319	2.57
Pharmaburst^®^ 500	29	111	208	1.61
Prosolv^®^ ODT G2	28	75	162	1.78
MIX	34	144	255	1.53

**Table IV:** Comparative Results for Co-Processed ODT Multifunctional Excipients and the Authors’ MIX

Excipient	Bulk density [mg/mL]	Tapped density [mg/mL]	Carr index	Hausner ratio	Angle of repose [°]	Flow through orifice [s]	True density [g/cm^3^]
F-Melt^®^ C	0.52 ± 0.02	0.60 ± 0.01	14 ± 1.3	1.16 ± 0.02	35 ± 1.3	3.0 ± 0.05	1.4842 ± 0.0008
F-Melt^®^ M	0.53 ± 0.03	0.61 ± 0.03	13 ± 0.5	1.15 ± 0.00	34 ± 2.5	3.6 ± 0.55	1.4862 ± 0.0005
Ludiflash^®^	0.54 ± 0.03	0.65 ± 0.01	17 ± 5.5	1.21 ± 0.08	39 ± 3.1	6.1 ± 1.48	1.4390 ± 0.0009
Pharmaburst^®^ 500	0.41 ± 0.03	0.47 ± 0.01	14 ± 4.4	1.16 ± 0.06	36 ± 1.5	4.2 ± 1.18	1.4131 ± 0.0007
Prosolv^®^ ODT G2	0.60 ± 0.01	0.74 ± 0.00	19 ± 0.5	1.24 ± 0.01	39 ± 1.3	9.4 ± 1.04	1.4776 ± 0.0005
MIX	0.43 ± 0.00	0.52 ± 0.01	17 ± 0.6	1.20 ± 0.01	41 ± 1.0	5.3 ± 0.33	1.4292 ± 0.0006

Pharmaburst^®^ 500 is composed of two types of particles, differing in size and morphology (Fig. 1D). The first one is represented by irregular aggregates of rough, lumpy surface, and a length not exceeding 100 µm across the long axis. The second type consists of solid particles of more spherical shape, smoother surface, and size varying between 60—200 µm. The observation stays in agreement with the laser diffraction data. Particle size distribution reveals two maxima, first in the vicinity of 30 µm, and the second, corresponding to a bigger particle population, at ca. 130 µm (Table III, Fig. S1). The excipient is characterized by good flow based on the Carr index and Hausner ratio, and fair flow according to the angle of repose test (Table IV).

Three types of particles were noticed in Prosolv^®^ ODT G2 (Fig. 1E). The first one is indicated by red arrows: smooth and spherical, with diameter of tens of micrometers. The second shown by green arrows are irregular, elongated aggregates of size varying between 50—200 µm. Finally, the third, marked by blue arrows look like thin platelets of length not exceeding 120 µm. The particle size distribution obtained with laser diffraction shows only one maximum, lying at the lowest value in comparison with other samples. The distribution is wide (span equal to 1.78, Table III) and tails towards small particles (Fig. S1). Prosolv^®^ ODT G2 particles are of the highest bulk and tapped density among the compared excipients. The Hausner ratio, compressibility index as well as angle of repose indicate fair flow of the powder (Table IV).

In the microphotograph of the MIX (Fig. 1F), particles of constituent excipients can be found. The most noticeable are spherical of smooth surface and diameter varying between 80—120 µm. They are typical for direct compressible grade of mannitol. Irregular, porous agglomerates are attributed to crospovidone. Some platelets of length below 100 µm are the most probably cellulose microcrystalline. Particle size distribution obtained in laser diffraction measurements confirms the presence of two particle populations, one corresponding to the maximum at around 140 µm, and the second, visible in the distribution tail, in the vicinity of 30 µm (Fig. S1). Flow characteristic is fair due to the compressibility index and Hausner ratio. The angle of repose indicates passable flow (Table IV).

#### Compaction Characteristics

**Fig. 2:**
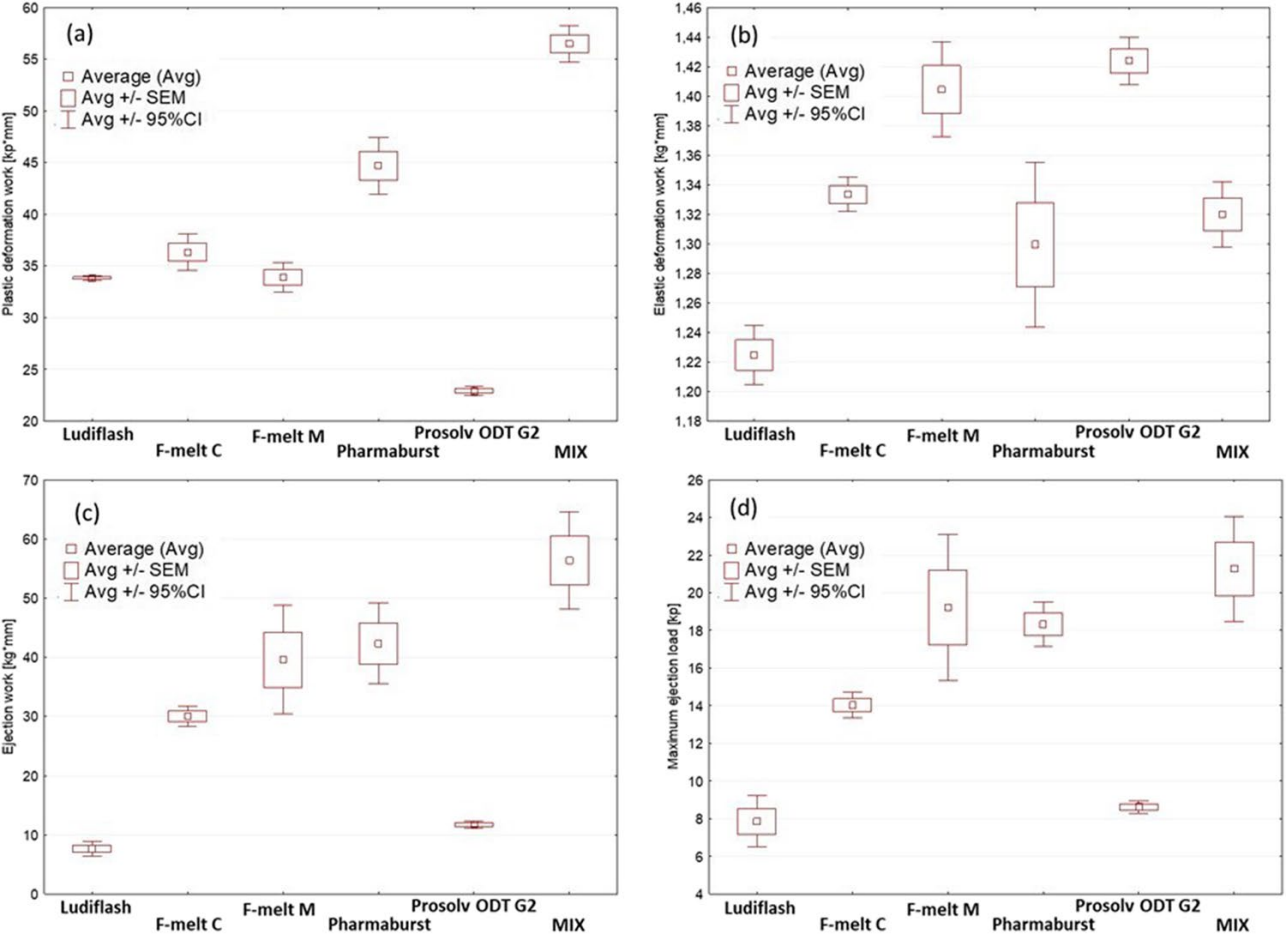
Evaluation of compaction simulator outcomes; (**a**) plastic work, (**b**) elastic work, (**c**) ejection work, and (**d**) max ejection load: average values, standard error of the mean (SEM) and 95% confidence intervals (CI).

The force–displacement-time profiles were established for each material. Area under the curve was subdivided into regions representing work involved in compression (plastic deformation), flow, decompression (elastic deformation) and tablet ejection. Results are presented in Fig. 2 and Table S1.

During the excipients testing, we recorded plastic deformation work (Fig. 2a, Table S1) in the range of 22.90—56.47 kp·mm. The highest values were obtained for the physical MIX. Results for Pharmaburst^®^ 500 were significantly lower (p < 0.001). The plastic deformation works for F-Melt^®^ C, F-Melt^®^ M, and Ludiflash^®^ were similar (*p* ≥ 0.4). The lowest value was recorded for Prosolv^®^ ODT G2 (*p* < 0.001). The area under the force–displacement curve, which is attributed to flow work, was exceptionally small for each tested excipient. The compared materials varied insignificantly. Elastic deformation work was recorded in the range of 1.23—1.42 kp·mm (Fig. 2b, Table S1). The highest values were measured for Prosolv^®^ ODT G2 and F-Melt^®^ M. Both were similar (*p* = 0.96). Lower values were recorded for F-Melt^®^C, physical MIX, and Pharmaburst^®^ 500. The all three are similar (*p* > 0.9). The elastic deformation work for the Ludiflash^®^ was significantly lower than the results obtained for all the other excipients (*p* < 0.05).

The ejection work in the range of 7.66—56.37 kp·mm is presented in Table S1. The highest values were noticed for the MIX. Ejection works for F-Melt^®^ M, F-Melt^®^ C, and Pharmaburst^®^ 500 were significantly lower in comparison to the physical MIX (*p* < 0.05) but similar to each other (*p* > 0.05). The lowest values were obtained for Ludiflash^®^ and Prosolv^®^ ODT G2. The latter two are similar (*p* = 0.9). Essentially similar trends were observed for maximal ejection load (Fig. 2d). The highest value was recorded for the MIX. The maximum ejection was similar to values obtained for F-Melt^®^ M (*p* = 0.8) and Pharmaburst^®^ 500 (*p* = 0.4). A significantly lower ejection load was obtained for F-Melt^®^ C. The lowest ones were noticed for Ludiflash^®^ and Prosolv^®^ ODT G2. The difference between the ejection forces recorded for the latter two is statistically non-significant (*p* = 0.9).

The ability to deform plastically under applied load is generally a desired feature in tablet manufacturing. Therefore, low work of plastic deformation favors the co-processed excipients like Prosolv_®_ ODT G2, next the both types of F-Melt^®^ and Ludiflash^®^. Significantly higher energy was needed for the plastic deformation of Pharmaburst^®^ 500 and the highest one during compaction of the MIX. The elastic recovery could weaken the forces binding particles into compacts. Since the tablet relaxation may cause capping and/or lamination, limited values of elastic work are desired. The relative recovery expressed as a percentage of elastic deformation work to plastic deformation work was ranging from ca. 2% as calculated for the physical MIX up to ca. 6% for the Prosolv_®_ ODT G2. Nevertheless, from a practical perspective, the results recorded for each material are considered low.

At the end of the compaction cycle, the tablet is removed from the die. The load applied vertically by the upper punch results in the powder’s horizontal expansion, wherefore the mass may adhere to the dies. The ejection work and force are proportional to the strength of binding between the side surface of the tablet and the die cavity. High ejection force can cause tablet defects and machine seizures. The highest values were recorded for the MIX. The finding looks consistent with the highest work of its plastic deformation. In order to prevent strong adherence of the particles to the die, addiction of sufficient quantity of lubricant is required.

The compared materials are of complex composition, i.e., contain excipients that behave differently under pressure. Microcrystalline cellulose is known as soft and ductile, subjected to plastic deformation with noticeable elastic recovery. Mannitol fragments under pressure and shows some plastic deformation at contact points [36]. Sorbitol and crospovidone are also plastically deformable. Granulated dibasic calcium phosphate (constituent of F-Melt^®^ C) is consolidated mainly through brittle fragmentation [31, 37–39].

The contribution of the constituent components was explained with so-called mesoscopic representation [36]. The model was developed for two ingredients admixed in 1:1 ratio. Each one was of significantly different ability for densification under pressure. The blends were compressed. As a result, volumetric representation of the less compressible material in the compact was much greater in comparison to the more compressible one. The ingredient that occupies larger relative volume plays dominant role in the whole compact’s behavior. Microcrystalline cellulose is the most compressible constituent, mannitol is of similar compressibility to the cellulose, and dibasic calcium phosphate densifies under pressure the least. Therefore, the mixtures containing cellulose and mannitol are expected to produce compacts of unchanged relative volumetric representation of each constituent. Both excipients are present in the MIX blend and Prosolv^®^ ODT G2. The F-Melt^® ^C contains among others co-processed cellulose microcrystalline and dibasic calcium phosphate, i.e., constituents of drastically different compressibility.

In the next stage, we compared the multicomponent excipients by performing manufacturability studies.

### Evaluation of Placebo Tablets

The co-processed multifunctional excipients as well as the physical blend (MIX) were admixed with 2% of sodium stearyl fumarate and compressed into ca. 500 mg tablets under 7 kN (66 MPa), 10 kN (88 MPa), and 12 kN (111 MPa). Eccentric Korsch EK0 equipped with round, flat punches of diameter 12 mm was used. Tablet mass and size were certainly too big for orodispersible tablets application but suitable for the raw materials comparison. Placebo dispersible tablets of the same weight and similar size were tested by Bowles *et al.* [31] for the excipients comparison. The compacts were tested for mass uniformity, resistance to crushing (hardness), thickness, friability, and disintegration time. Results are given in Table S2 in Suplementary Information.

The weight uniformity of tablets containing co-processed excipients was within the range: average ± 1%. The physical MIX produced tablets of a bit poorer mass uniformity, i.e., all the results were distributed up to 2% out of the average.

**Fig. 3:**
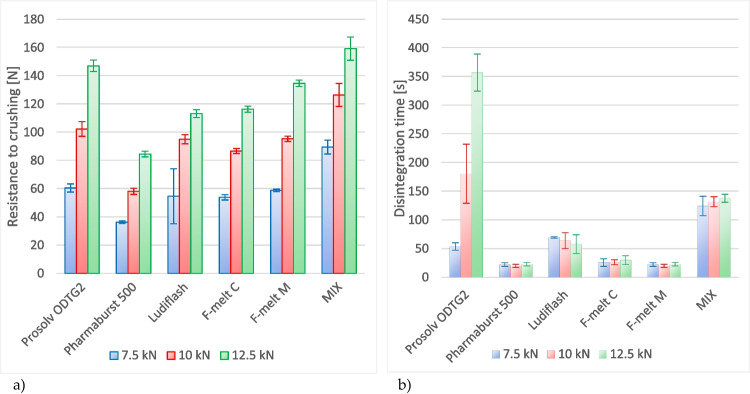
Resistance to crushing (breaking force, hardness) and disintegration time of tablets compressed under load 7.5 kN, 10 kN and 12.5 kN. The center points indicate averages, the error bars are standard deviations. The lines combining the centre points are to facilitate the results comparison only (do not have any physical meaning).

Tablets’ resistance to crushing as a function of compression force is called manufacturability. For each excipient, tablet breaking force raised up with increasing the compression load. There are differences in the sensitivity of the materials to compression force. The highest breaking force characterizes the tablets containing MIX, while the lowest one the Pharmaburst^®^ (Fig. 3). Use of Prosolv^®^ ODT G2, F-melt^®^ M, Ludiflash^®^, and F-melt^® ^C resulted in tablets of similar breaking force when compressed with forces in range 7.5—10 kN. The highest load of 12.5 kN differentiated the four excipients in the following order of descending breaking force: Prosolv^®^ ODT G2, F-Melt^®^ M, Ludiflash^®^ and F-Melt^® ^C.

Friability is a measure describing mechanical strength of compacts, which is supplemental to the tablet breaking force. It expresses the mechanical resistance of tablets against abrasion, which can occur during packaging, transportation, or removal from unit packs by patients. The feature shows the superiority of co-processed F-Melts^®^, which were characterized by outstandingly low friability within the full range of compression loads. The results are practically the same for both C and M grades, although tablets containing the M grade were of significantly higher hardness (p < 0.05) while pressed with 10 kN and 12.5 kN force. The F-Melt^®^ M is known for its better compactibility in comparison to the F-Melt^®^ C [14]. Next in the friability row was Prosolv^®^ ODT G2, although the hardness of these tablets was significantly higher in comparison to compacts made of the F-Melts^®^ (p < 0.05 for 10 kN load and p < 0.01 for 12.5 kN). The three excipients mentioned above delivered tablets of friability below 1.0%, regardless of the applied compression force. Tablets containing the physical MIX were of borderline friability, i.e., 1.2%, 1.0%, and 0.9%, respectively, in the series of increasing compression force. Nonetheless, the hardness of tablets containing MIX was the highest among the whole group. The last positions in the friability order are taken by Ludiflash^®^ and Pharmaburst^®^ 500. The results were distinctly above 1%, reaching even 2% while tablets were pressed with a 7.5 kN load. The hardness of Ludiflash^®^ tablets compressed with 7.5 kN was similar to Prosolv^®^ and both F-Melts^®^. When higher loads were applied, the hardness of Ludiflash^®^ tablets was lower than that of the ones made of Prosolv^®^ but still similar, i.e. with statistically non-significant differences to F-Melt^®^ M (10 kN) or F-Melt^®^ C (12.5 kN).

Disintegration time is a very important attribute, especially for orodispersible tablets. According to Ph. Eur., the ODTs should disintegrate within 3 min when placed into water at 37°C, and the basket-rack assembly should be equipped with discs. However, the FDA’s stringent acceptance criterion goes down to 30 s [40]. The shortest disintegration time was recorded for Pharmaburst^® ^500 and F-Melt^® ^M tablets (20—23 s) – see Fig. 3. Note that the numbers presented in brackets are the averages for the minimum and maximum applied compression force, respectively. Disintegration times noticed for the F-Melt^® ^C tablets were only slightly longer (26—30 s). Although the placebo tablets of exceptionally big size and mass (12 mm in dia., 500 mg) were investigated, the formulas composed of Pharmaburst^®^ 500 and both F-Melts^®^ were very close to the criterion recommended by the FDA [40]. All the other samples were in line with Ph. Eur. requirements only. Next in the row were Ludiflash^®^ (70—58 s), the MIX (124—138 s), and Prosolv^®^ ODT G2 (54—356 s). The Prosolv^®^ is especially sensitive to higher compression forces: the disintegration time under a load of 7.5 kN is shorter than 1 min while applying 12.5 kN prolongs it up to almost 6 min. Similar results were described by Brniak *et al.* [33], who compared 400 mg placebo tablets of diameter 12 mm containing F-Melt^®^ C, Pharmaburst^®^ and Ludiflash^®^, admixed with 1% and 2% of sodium stearyl fumarate. The authors tested disintegration time using three methods, i.e., pharmacopoeial, innovative one utilizing their own-designed apparatus and in oral cavity. The advantage of the F-Melt^®^ C was shown by a comparative disintegration test performed in the innovative apparatus and pharmacopoeial one. In the oral cavity test, all the three ODT formulas disintegrated within 1 min. However, Ludiflash_®_ was the most effective: tablets compressed with forces in the range of 10—20 kN disintegrated within 30 s. Pharmaburst^®^ formula was not considered, since tablets were mechanically too weak. Similar results were obtained in the case of the currently described results; tablets with this excipient exhibited the worst mechanical properties as given by the highest friability.

According to USP-NF < 1062 > , we established tabletability profile, which is a function of tablet tensile strength *vs.* compression pressure. Thanks to that, the impact of tablet size, thickness and weight on the data analysis was minimized. Furthermore, we measured true density of the co-processed excipients and the MIX using a gas pycnometry (Table IV). Based on the results, we calculated solid fraction for the tablets compressed under various pressures. The solid fraction known as a relative density is a measure of the volume of solid material in a compact [32]. The characteristics were used for the establishment of powder compressibility and compactibility. Compressibility is the dependence of tablet solid fraction on compression pressure, while compactibility is a relationship between tensile strength and the solid fraction. It is assumed that the desired solid fraction for tablets is within a range of 0.80—0.90 [31]. Therefore, in order to compare diverse materials, we listed compression pressures needed to achieve the compacts of the reference solid fraction of 0.85 [31] and tensile strengths of produced tablets (Table V).

**Table V:** Tablet Compression Profiles Established for Tablets of Solid Fraction Equal to 0.85

Excipient	Compression pressure [MPa]	Tensile strength [MPa]	Compression pressure to tensile strength ratio
F-Melt C^®^	109	1.74	62.6
F-Melt M^®^	112	2.07	54.1
Ludiflash^®^	104	1.61	64.6
Pharmaburst^®^ 500	182	2.16	84.3
Prosolv^®^ ODT G2	99	1.81	54.7
MIX	142	3.17	44.8

In order to achieve compacts of a solid fraction equal to 0.85, we can distinguish three compression pressure levels: the highest one needed for Pharmaburst^®^ 500, markedly lower for the MIX, and the lowest one for the rest of the co-processed excipients (Table V). The materials can be ordered by increasing compressibility as follows: Pharmaburst^®^ 500 < MIX < F-Melt^®^ M < F-Melt^®^ C ≤ Ludiflash^®^ < Prosolv^®^ ODT G2. It means that in order to produce tablets of desired porosity, the smallest load is required for the compression of Prosolv^®^ ODT G2 [32, 37]. The results stay in agreement with plastic deformation work recorded with a Gamlen compaction simulator. The highest work was needed for the deformation of MIX and Pharmaburst^®^, and the lowest for Prosolv^®^ ODT G2. The rest of the excipients were at a similar level. It occurs that significant energy was utilized to overcome resistance during particle compression. Therefore, the most compressible material (Prosolv^®^ ODT G2) required the lowest work. Knowing the compression pressure needed for each powder, we compared the tensile strengths of the compacts. The function is called tabletability. In order to compare the ingredients, we calculated the compression pressure to tensile strength ratio (Table V). A low factor indicates materials of good tabletability because applied pressure causes high tensile strength of compacts (high value of denominator). Given the calculated ratios, one can conclude that in order to obtain tablets of given tensile strength, the highest load will be needed for Pharmaburst^®^ 500 and the lowest for the MIX.

The next characteristic taken into consideration is compactibility. The relationship between tensile strength and solid fraction describes the strength of bindings created between adjacent plastically deformed particles. Having the same solid fraction for each compacted excipient, the excipients can be ordered according to the highest tensile strength as follows (Table V): MIX > Pharmaburst^®^ > F-Melt^®^M > Prosolv^®^ODTG2 > F-Melt^® ^C > Ludiflash^®^. Assuming that the powders are consolidated to the same extent, the strongest bindings join particles of the physical MIX and the weakest the Ludiflash^®^. Differentiation in compressibility, tabletability, and compactibility, once again expresses the complexity of the composition and preparation ways of the compared materials. Considering the Pharmaburst^®^, the highest pressure is required for the material compression up to 0.85 solid fraction. Having that density of compacts, the particle bindings are quite strong but not the strongest. On the contrary, the weakest bindings are attributed to Ludiflash^®^; however, compression of this material needed one of the lowest force. Ludiflash^®^ appears to us really interesting due to markedly low works of plastic deformation, elastic recovery, and ejection.

From the perspective of ODT manufacturing it is not obvious which is better: sensitiveness or resistance to pressure. Material that does not significantly change its properties when subjected to increasing load may create quality ODTs within a wide range of tableting parameters, like compression forces and dwell times. Such property may be considered as robustness. Therefore, the tabletability, compressibility, and compactibility is evaluated in parallel to the other attributes, especially friability and disintegration time.

Given the analyzed excipients, the friability results look consistent with tabletability and compactibility of the materials. The only exception is the physical MIX, which was characterized by the highest compactibility but only moderate friability. The friability, as well as compactibility, seem to give valuable information about the practical aspects of tablet performance. High resistance to crushing was not necessarily attributed to good quality since high-hardness tablets tend to be friable (e.g., the MIX). A poor correlation between friability and crushing strength is reported in the literature [23].

Bowles *et al.* [31] tested several excipients, including the F-Melts^®^ C and M, Pharmaburst^®^ 500, and Ludiflash^®^. The experiments aimed to evaluate the suitability of materials for the preparation of dispersible tablets via direct compression method. The Authors assumed that drug substances are usually poorly compressible, and therefore, they specified two levels of acceptance criteria—ideal specification and minimal requirements. Assessing manufacturability the following outcomes and the requirements (in brackets) were applied: powder flow (Carr index ideally < 15%, min. < 20%), tensile strength at 0.85 solid fraction (≥ 3.0 MPa, min. ≥ 1.5 MPa), ejection shear (< 3.0 MPa, min. < 5.0 MPa), friability (ideal < 1% in 10 min, min. < 1 in 4 min), disintegration time (< 60 s, min. < 180 s) and fineness of dispersion (ideal passes through 0.25 mm sieve, min. passes through 0.71 mm). The materials were lubricated with 1% of sodium stearyl fumarate and pressed into 500 mg tablets. Among 17 excipients, the F-Melt^®^ C was rated as the best performing (among two others not commented on here). The score is based on tensile strength > 3 MPa, ejection shear ca. 0.7 MPa, very low friability (0.02% after 4 min and 0.12% after 10 min), disintegration time ca. 40 s, and fine dispersion after reconstitution in water. F-Melt^®^ M, Pharmaburst^®^ 500 and Ludiflash^®^ were distinguished as appropriate for the preparation of the dispersible tablets.

Another comprehensive comparison is given by Stoltenberg and Breitkreutz [23]. However, the investigation was focused on mini ODTs that are of mass ca. 6.5 mg and diameter of 2 mm. Properties of five co-processed excipients including Ludiflash^®^, Prosolv^®^ ODT, and Pharmaburst^®^ 500 were compared. The powder blends containing 15.4% of hydrochlorothiazide and 3.5% of sodium stearyl fumarate were compressed into mini-tablets of crushing strength of ca. 7 N. As a control, placebo formulations were tested. Among the groups, superior properties were found for the Ludiflash^®^ due to high crushing strength, low simulated wetting time, low friability, and good flow. Nevertheless, all the excipients were considered suitable for manufacturing the mini-ODTs. The small size of investigated compacts made the comparison between the cited paper and our work difficult. Also, it revealed some opposite results, e.g., high crushing strength noticed for Pharmaburst^®^ 500 formula, which was inconsistent with our findings.

### Machine Speed Sensitivity Study—Evaluation of Bromhexine Hydrochloride Tablets

In the next step, we performed the machine speed sensitivity study by using Korsch PH103 rotary press. The formulas were composed of the investigated excipients, bromhexine hydrochloride (8 mg), sucralose (2 mg), and lemon flavor (2 mg) (Table II). Round, flat punches of diameter 7 mm were designated for tablets of mass of 100 mg. The Korsch PH103 was not instrumented, therefore we were not able to control compression force. At the beginning of the tableting of a particular batch, the machine operated at lower speed (38 rpm was set with the load sufficient to obtain tablets of satisfactory friability). After the sample of tablets was pooled, the higher speed was set without correction of the tablet mass and any change in load. Then, another sample of tablets was collected for testing.

The most significant drop in hardness was noticed for F-Melt^® ^M, Prosolv^®^ ODT G2 and Pharmaburst^®^ 500 (Table VI). However, the results were correlated with a drop of tablet average mass, i.e., 5.5 mg in case of F-Melt^®^ M, 2.6 mg for Prosolv^®^ ODT G2, and 3.1 mg for Pharmaburst^®^ 500. Hardness of tablets containing the other materials slightly increased when the compression speed was set at a higher level. The fact is also explained by a slight increase in the tablets’ average mass. The Korsch PH103 machine operated at 38 rpm compress powders with a dwell time of 70.4 ms, when at 76 rpm with 35.2 ms. A shorter dwell time promotes lower tablet hardness. Therefore, the increase in tablet hardness can be explained only by an increase in powder volume in the die. More powder means that a higher pressure is exerted when punches upper and lower are coming into the closest distance. All the tested tablets were of good mechanical strength expressed by friability below 0.6%. Performance of the F-Melts^®^ looks outstanding: friability for F-Melt^®^ C tablets was 0.1% (both speeds of turret), and for F-Melt^®^ M tablets was 0.2% at 38 rpm and 0.3% at 76 rpm.

**Table VI:** Properties of Tablets Containing Bromhexine Hydrochloride

Results: mean ± standard deviation												
	Pharmaburst^®^ 500		Prosolv^® ^ODT G2		F-Melt^® ^C		F-Melt^® ^M		Ludiflash^®^		MIX	
Tableting speed [rpm]	38	76	38	76	38	76	38	76	38	76	38	76
Mass [mg] *N* = 10	104.1 ± 0.74	101.0 ± 1.05	103.0 ± 0.94	101.9 ± 1.10	96.5 ± 1.72	99.3 ± 0.95	104.9 ± 0.88	99.4 ± 0.70	100.6 ± 2.01	103.7 ± 2.36	97.6 ± 2.41	102.4 ± 2.12
Hardness [N] *N* = 10	28.9 ± 3.50	19.9 ± 3.43	21.7 ± 1.08	8.6 ± 0.77	22.2 ± 0.51	25.7 ± 0.77	28.2 ± 1.31	14.5 ± 1.90	21.1 ± 3.37	23.1 ± 1.25	15.9 ± 0.62	18.8 ± 0.77
Friability [%]	0.3	0.6	0.1	0.4	0.1	0.1	0.2	0.3	0.5	0.6	0.4	0.4
Disintegration time [s] *N* = 6	5 ± 1.03	5 ± 1.03	100 ± 16.02	10 ± 1.79	11 ± 3.27	24 ± 6.12	6 ± 0.82	28 ± 6.20	18 ± 3.20	28 ± 3.88	6 ± 1.79	8 ± 1.51
Content [%] *N* = 6	103 ± 0.29	-/-	102 ± 1.95	-/-	103 ± 1.27	-/-	100 ± 3.18	-/-	103 ± 1.81	-/-	101 ± 2.92	-/-

Content uniformity results indicate an even distribution of bromhexine hydrochloride within all the powder blends (RSD in the range of 0.3—3.2%). The average disintegration time was shorter than 30 s for almost all tested samples. The only exception was the formula containing Prosolv^®^ ODT G2 pressed at a lower speed. The average disintegration time was 1 min 40 s and the maximum 1 min 56 s. However, after an increase in the turret speed, the disintegration time dropped drastically down to avg. of 10 s (max. 12 s).

**Fig. 4:**
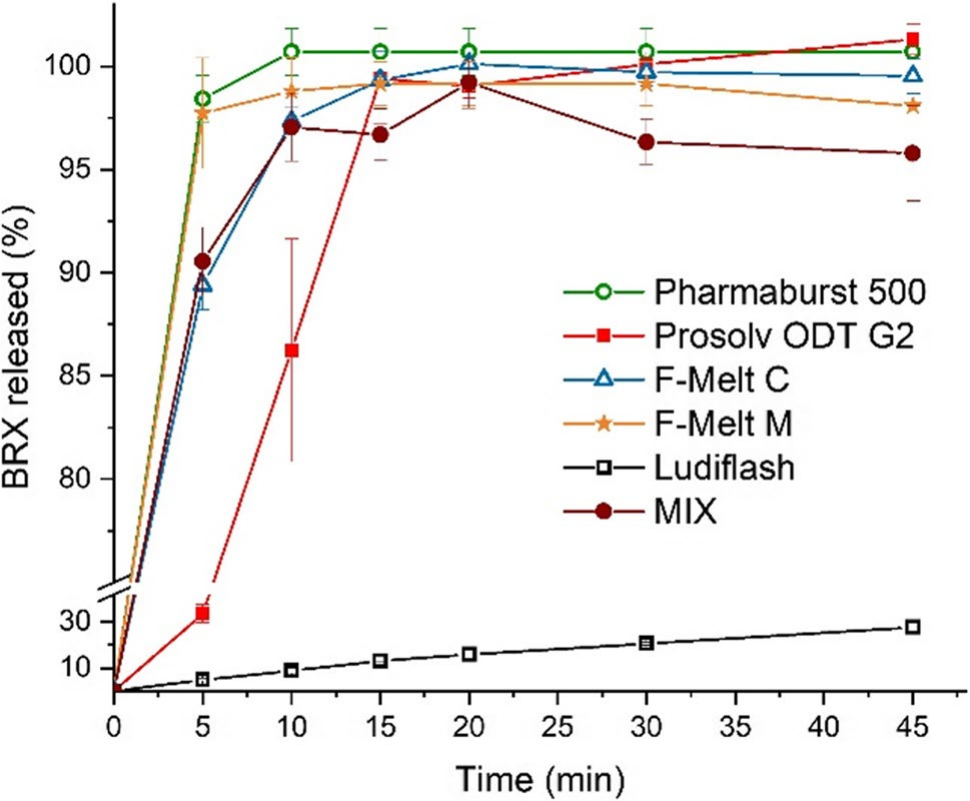
Dissolution profiles for ODTs of composition given in Table II. Test performed in 900 mL of 0.1 M HCl, Ph. Eur. app. 2 operated at 50 rpm.

Dissolution of the bromhexine hydrochloride was tested for tablets compressed at a lower speed, i.e., at longer dwell time (the worst-case scenario). In 900 mL of 0.1 mol/L solution of hydrochloric acid, dissolution was fast and complete for all the formulations except the one containing Ludiflash^®^ (Fig. 4). We have not found signs of interaction between the active and Ludiflash^®^ in tests performed with DSC and HPLC methods (data not presented here). Similar phenomenon was observed by Tayel *et al.*, who tested various formulae of sumatriptan succinate sublingual tablets [19]. The notably prolonged dissolution of Ludiflash^®^ based formulae was attributed to the presence of Kollicoat SR30D, which is used to prepare sustained-release dosage forms. Another work, by Raykar and Velraj, reported tablets containing tofacitinib citrate, Ludiflash^®^, and sodium starch glycolate [41]. Among the nine formulae tested, seven released less than 60% of the drug within 30 min, and only one released the entire dose. Contrary data was reported by the BASF company [42], which showed that tablets containing 1% of risperidone and 96% of Ludiflash^®^ or 2% of loperamide and ca. 95% of Ludiflash^®^ exhibited complete drug dissolution after 30 min of testing.

To sum up, all the formulations tested seem suitable to manufacture tablets of uniform mass, and the active ingredient content, low friability, and disintegration time that complies with compendial criteria. The self-prepared formulation (MIX) was comparable with the co-processed excipients and therefore selected for transfer to the production scale.

### Scale-Up Results and Biopharmaceutical Aspects

**Fig. 5:**
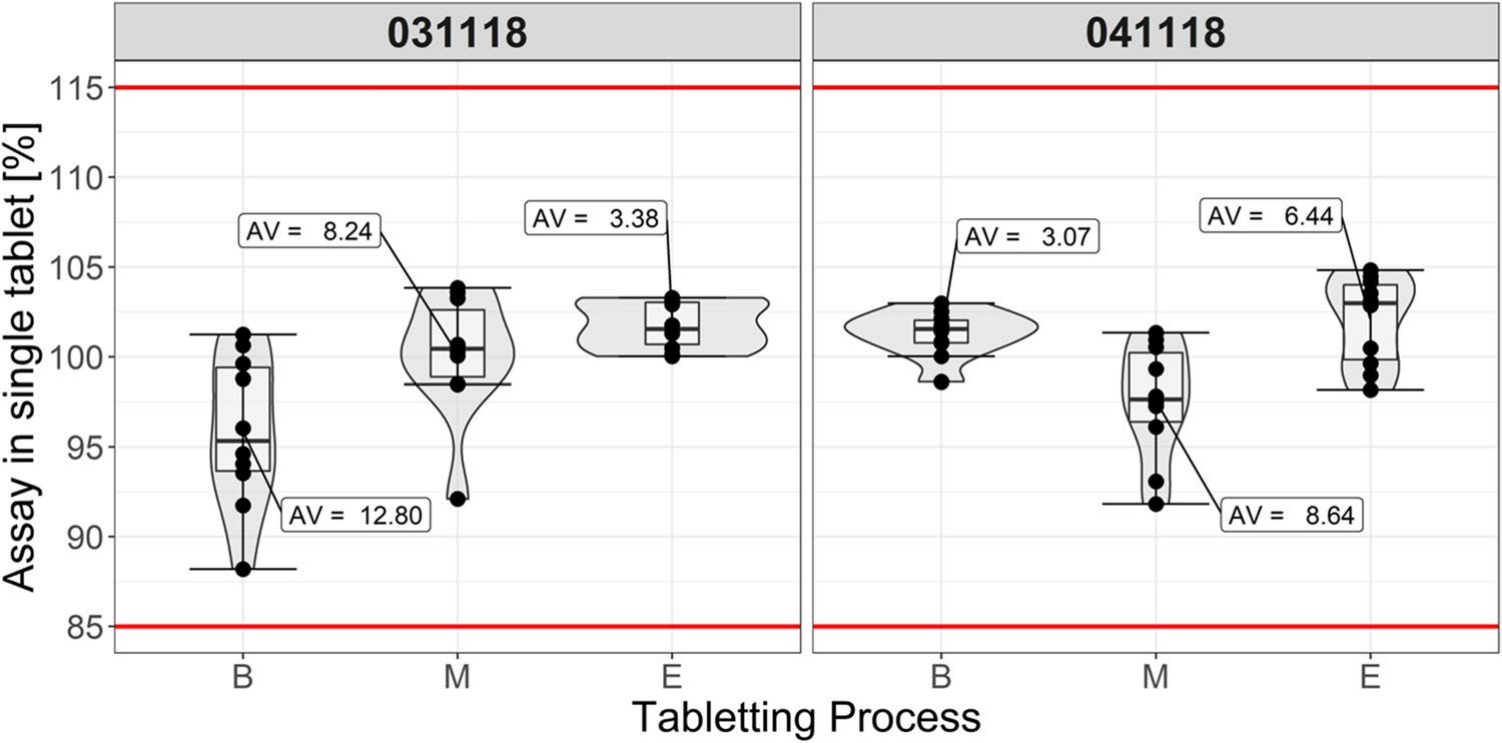
Content uniformity variation during compression process. Samples were collected at the beginning (B), in the middle (M), and the end (E) of tableting.

The formula containing direct-compressible excipients (MIX) was blended with 8 mg of bromhexine hydrochloride, lubricant, and compressed in production scale. The tableting process went smoothly. The outcomes are presented in Table VII and Fig. 5. Tablets exhibited uniform mass and short disintegration time. Both batches were of sufficient hardness (ca. 40 N) and very low friability. The tensile strengths calculated for the two lots were 1.5 MPa and 1.7 MPa, respectively. It is assumed that tensile strength not less than 1.7 MPa ensures that tablets will not be damaged during packaging and distribution. For small batches, the criterion is lowered to 1 MPa [43]. In general, the range of 1—2 MPa is common for marketed products [44]. We have not noticed any problem with the mechanical resistance of our product during transportation in barrels and subsequent packaging. Amount of the bromhexine hydrochloride determined is found almost equal to the label claim.

**Table VII:** Attributes of BRX Tablets Obtained in Production Scale

Quality attribute	First lot	Second lot
Appearance	White to off white, round, flat tablets	
Tablet mass (range) [mg]	102.0 (99—103)	101.5 (100—103)
Resistance to crushing [N]	36.7	42.7
Thickness [mm]	2.28	2.26
Tensile strength [MPa]	1.46	1.72
Friability [%]	0.1	0.3
Disintegration time [s]	10	12
Assay of bromhexine HCl	99.8% of label claim	99.6% of label claim
Loss On Drying [%]	1.6	1.2
Yield [%]	92.5	93.5

The tableting process was well responsive to tablet weight regulations seen as local increase of AV followed by steady and well centered position of the next stage (Fig. 5).

As highlighted by CPMP/EWP/QWP/1401/98 regarding orodispersible tablets, “placement in the mouth and time of contact may be critical in cases where the active substance is dissolved in the mouth and can be absorbed directly via the buccal mucosa”. To verify whether the active ingredient dissolves in the mouth, the disintegration time of tablets and the active substance release rate were studied under the conditions simulating the oral cavity. The results for the first lot confirmed that the disintegration of developed ODTs is rapid, regardless of the measurement conditions, i.e., 18 s in the Ph. Eur. apparatus and 27 s in the BJKSN-13 apparatus. The dissolution of bromhexine hydrochloride under simulated oral conditions reached only 0.62% of the 8 mg dose (SD = 0.15) after 5 min and 1.28% (SD = 0.28) after 10 min. The data confirms that the dissolution of the active substance under conditions that mimic oral cavity is negligible, which results from the physicochemical characteristics of the drug substance. Bromhexine is a moderately strong base (pK_a_ = 9.2), so the compound tends to dissolve in acidic media, while in the alkaline environment, it remains mostly in the non-ionized form [45]. In our opinion the orodispersible tablets will disintegrate rapidly in saliva, but the drug substance will dissolve mainly in the stomach or duodenum.

**Fig. 6:**
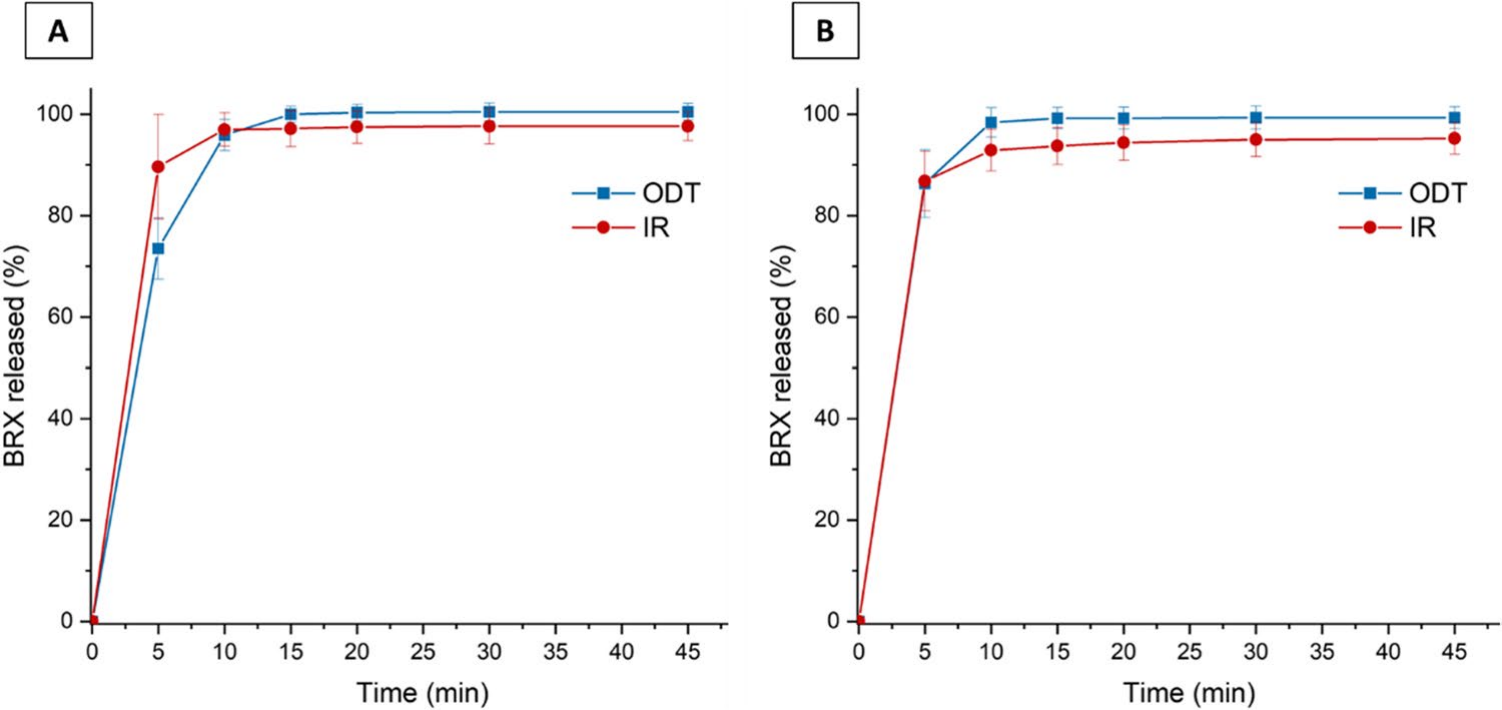
Dissolution profiles for the first lot of ODT and the immediate release product (IR) available on the market (Bisolvon 8 mg tablets manufactured by Boehringer Ingelheim). The error bars indicate standard deviation. Test was performed in 900 mL of 0.1 M HCl (**A**) and phosphate buffer of pH 4.5 (**B**) with Ph. Eur. app. 2 operated at 50 rpm.

The subsequent studies aimed to check the release rate of the bromhexine hydrochloride from ODTs in the media representing the upper part of the gastrointestinal tract. Based on solubility studies performed at 37°C, we found that the following volumes of media are needed to dissolve the highest strength of the drug substance: 54 mL of water, 48 mL of 0.1 mol/L solution of hydrochloric acid and a phosphate buffer with pH = 4.5. The most challenging dose/solubility ratio was found in pH = 6.8 phosphate buffer, resulting in almost 2400 mL. The poor solubility in medium of the neutral pH explains the lack of drug dissolution in simulated saliva. On the other hand, good solubility in water and in media of pH = 1—4.5 indicates that the drug product will dissolve shortly in the stomach and should be quickly accessible for absorption. The dissolution trials performed in 0.1 mol/L hydrochloric acid solution and buffer of pH = 4.5 showed practically no difference between the most known product in form of immediate-release tablets (IR) and the ODTs (Fig. 6).

Assuming the lack of drug absorption in the oral cavity caused by very low solubility in neutral media, fast and complete dissolution in media simulating stomach and duodenum, similarity of dissolution profiles to the marketed IR tablets, favourable safety profile of bromhexine hydrochloride and well-established history in treatment, our ODT product is considered therapeutically equivalent to the conventional drug. A general recommendation is to perform a 3-period bioequivalence study in case the ODT drug is an extension to another, e.g., immediate release formulation [24, 25]. The regulatory biowaiver could be justified based on biopharmaceutical classification and is limited to well-soluble active ingredients only. Although low solubility of bromhexine hydrochloride in neutral media excludes it from the BCS I class, in our opinion registration of the product in line with the Well-Established-Use procedure without bioequivalence testing should be possible.

## Conclusions

We have performed experiments aimed to investigate the effect of commercially available multifunctional co-processed excipients, i.e., F-Melt^®^ C, F-Melt^®^ M, Ludiflash^®^, Pharmaburst^®^ 500, Prosolv^®^ ODT G2, and self-prepared physical blend of directly compressible excipients on the attributes of orodispersible tablets containing 8 mg of bromhexine hydrochloride. We compared powder flow, true density, compaction characteristics, and tableting speed sensitivity. The manufacturability studies confirmed that all co-processed excipients were very effective as the ODT formula constituents. We noticed superior properties of the F-Melt^®^ C and F, i.e., good mechanical strength of tablets and very competitive disintegration time. Also, the Ludiflash^®^ is outstanding due to low works of plastic deformation, elastic recovery, and ejection. The only doubt related to tablets containing Ludiflash^®^ was slow dissolution of active substance. The self-prepared blend of excipients possessed satisfying properties (high compactibility, acceptable friability, short disintegration time), thus, it was selected to transfer to the production scale. The outcome of the scale-up trial fully confirmed previous findings. Tablets with BHX complied with compendial requirements related to mass and content uniformity, assay, disintegration time, and dissolution. The results revealed that the dissolution of bromhexine hydrochloride under conditions mimicking the oral cavity is negligible. However, good solubility in acidic media indicates that the drug product will dissolve shortly in the stomach and as is should be quickly accessible for absorption. We proved that the dissolution profiles in media representing the upper part of the gastrointestinal tract are similar to marketed drug product. In our opinion, the developed formula is suitable for registration within the well-established use procedure without the necessity of bioequivalence testing.

## Patents

Patent application no. WIPO ST 10/C PL441842.

### Supplementary Information

ESM 1 (PDF 547 kb)

## Data Availability

The datasets generated during and/or analysed during the current study are available from the corresponding author on reasonable request.
