# Supplementary material for: Investigating the Impact of Co-processed Excipients on the Formulation of Bromhexine Hydrochloride Orally Disintegrating Tablets (ODTs)
Source: Pharm Res. 2023 Sep 19;40(12):2947–62. doi: 10.1007/s11095-023-03605-x (PMC10746752; doi:10.1007/s11095-023-03605-x)
Supplement: Supplementary file 1 — (PDF 547 kb) [file 11095_2023_3605_MOESM1_ESM.pdf]

Supplemental Material

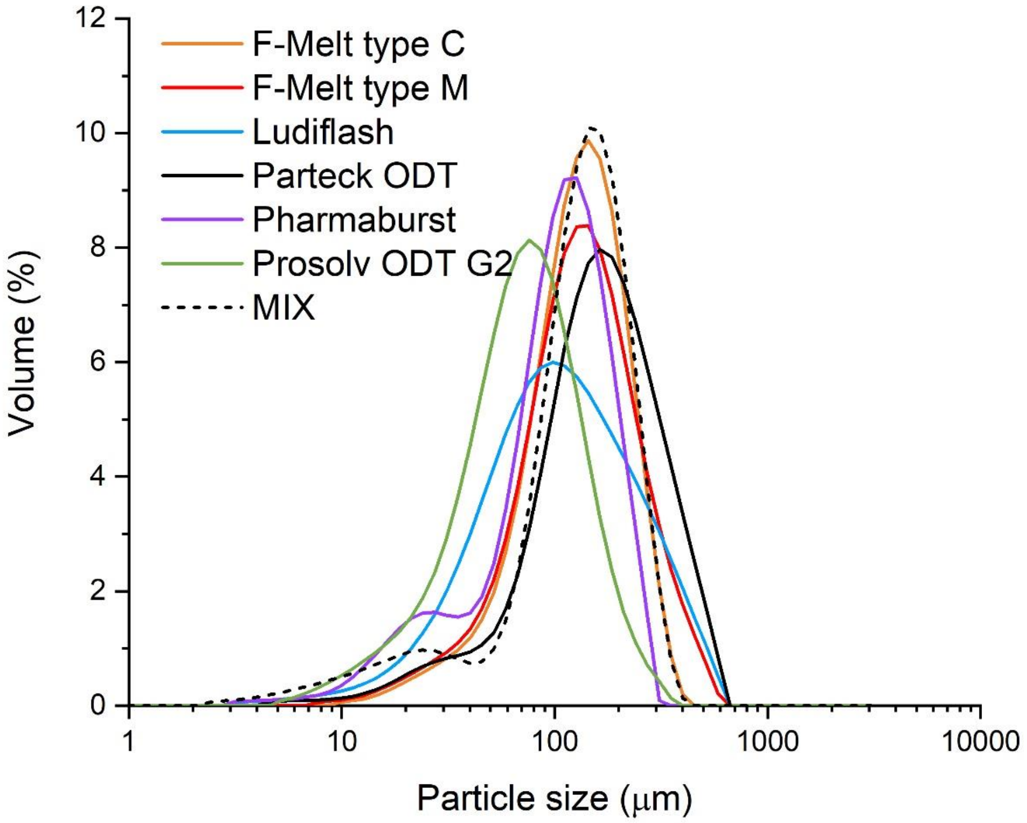

Figure S1. Particle size distributions of F-Melt type C, F-Melt type M, Ludiflash, Pharmaburst 500, Prosolv ODT G2, and MIX.

Table S1. Outcomes of measurements performed by using compaction simulator.

**Substance**	**Plastic work** [kp·mm]	**Elastic work** [kp·mm]	**Ejection work** [kp·mm]	**Max ejection load** [kp]
	Avg ± SD	Avg ± SD	Avg ± SD	Avg ± SD
F-Melt® C	36.33 ± 2.38	1.33 ± 0.02	30.05 ± 2.39	14.03 ± 0.91
F-Melt® M	33.89 ± 1.79	1.40 ± 0.04	39.57 ± 11.47	19.23 ± 4.84
Ludiflash®	33.83 ± 0.31	1.23 ± 0.03	7.66 ± 1.68	7.85 ± 1.84
Pharmaburst® 500	44.69 ± 3.68	1.30 ± 0.08	42.37 ± 9.22	18.34 ± 1.58

Prosolv® ODT G2	22.90 ± 0.85	1.42 ± 0.03	11.72 ± 1.06	8.61 ± 0.64
Author's mix	56.47 ± 2.37	1.32 ± 0.03	56.37± 11.08	21.27 ± 3.76

*Avg – average, SD – standard deviation

Table S2. Properties of placebo tablets obtained at 7.5 kN, 10 kN and 12.5 kN compression force.

**Name**	**Mass [mg]** *N = 10*	**Hardness [N]** *N = 10*	**Thickness [mm]** *N = 10*	**Tensile Strength [MPa]** *N = 10*	**Disintegration time [s]** *N = 6*	**Friability [%]**
	7.5 kN (66 MPa) results: **Average** ± SD*					
F-Melt® C	**497.6** ±1.17	**53.8** ±1.84	**3.786** ±0.008	**0.75** ±0.027	**26** ±6.6	**0.7**
F-Melt® M	**489.8** ±4.32	**58.7** ±0.90	**3.843** ±0.009	**0.81** ±0.013	**22** ±3.2	**0.7**
Ludiflash®	**501.4** ±1.90	**60.6** ±3.17	**3.801** ±0.016	**0.85** ±0.044	**70** ±1.3	**1.8**
Pharmaburst® 500	**505.2** ±1.13	**36.3** ±0.92	**4.403** ±0.024	**0.44** ±0.012	**22** ±3.2	**2.0**
Prosolv® ODT G2	**494.2** ±2.35	**60.3** ±2.86	**3.760** ±0.014	**0.85** ±0.040	**54** ±6.4	**1.0**
Mix	**490.3** ±4.37	**89.4** ±4.93	**4.061** ±0.035	**1.17** ±0.060	**124** ±16.7	**1.2**
	10 kN (88 MPa) results: **Average** ± SD*					
F-Melt® C	**498.1** ±0.99	**86.5** ±1.78	3.607 ±0.013	**1.27** ±0.028	**26** ±4.3	**0.4**
F-Melt® M	**492.2** ±1.23	**95.2** ±1.91	3.637 ±0.007	**1.39** ±0.029	**20** ±2.8	**0.5**
Ludiflash®	**502.5** ±2.17	**94.9** ±3.21	3.621 ±0.014	**1.39** ±0.046	**64** ±13.9	**1.3**
Pharmaburst® 500	**505.7** ±1.49	**58.0** ±2.19	4.203 ±0.020	**0.73** ±0.030	**20** ±2.8	**1.3**
Prosolv® ODT G2	**495.4** ±2.63	**102.1** ±5.22	3.547 ±0.018	**1.53** ±0.080	**180** ±51.3	**0.5**
Mix	**477.5** ±5.08	**126.3** ±8.17	3.717 ±0.014	**1.80** ±0.111	**132** ±8.3	**1.0**
	12.5 kN (111 MPa) results: **Average** ± SD*					
F-Melt® C	**498.5** ±1.08	**116.2** ±2.18	3.481	**1.77** ±0.038	30 ±7.8	**0.3**
F-Melt® M	**492.3** ±0.67	**134.5** ±2.29	3.480	**2.05** ±0.036	23 ±3.0	**0.3**
Ludiflash®	**500.7** ±3.06	**113.0** ±2.81	3.513	**1.71** ±0.038	58 ±16.3	**1.3**
Pharmaburst® 500	**505.9** ±0.99	**84.4** ±2.02	4.036	**1.11** ±0.032	23 ±3.0	**1.1**
Prosolv® ODT G2	**494.6** ±2.12	**146.9** ±3.95	3.415	**2.28** ±0.064	356 ±32.2	**0.4**
Mix	**473.4** ±4.97	**158.9** ±8.25	3.590	**2.35** ±0.118	138 ±6.7	**0.9**

* friability test was performed for each formulation only once (N = 1)
